# Effect of Mental Health Care Visits on HIV Care Outcomes

**DOI:** 10.1007/s10461-024-04542-5

**Published:** 2024-11-08

**Authors:** Morgan E. Bussard, Sunbal Ashraf, Nathan A. Summers

**Affiliations:** 1 College of Medicine, University of Tennessee Health Science Center College of Medicine, Memphis, TN USA; 2 Department of Psychiatry, University of Tennessee Health Science Center, Memphis, TN USA; 3 Adult Special Care Center, Regional One Health, 880 Madison Ave., Memphis, TN USA; 4 Department of Medicine, Division of Infectious Diseases, University of Tennessee Health Science Center, Memphis, TN USA

**Keywords:** HIV care cascade, viral suppression, mental health care

## Abstract

To improve the quality of life for people living with HIV (PLWH), it is vital their treatment plans closely follow the HIV care continuum. However, many barriers, such as mental health disorders (MHD), can complicate treatment. Patients being treated for HIV with comorbid MHD are more likely to not be retained in care and maintain an unsuppressed viral load. As PLWH and people vulnerable to acquiring HIV are more commonly diagnosed with MHD in comparison to the general population, it is important that steps to mitigate the possible effects of MHD are addressed during treatment. This study examines how minimal mental health care in a safety-net hospital system in the U.S. South can show benefits in retaining patients throughout their treatment of HIV. The results showed that older individuals retained a higher level of viral suppression when they followed up regularly with a mental health care provider during treatment. In addition, regardless of age, the higher the number of mental health care visits a patient attended during treatment, the higher the likelihood of viral suppression. By incorporating mental health care into the HIV treatment plan, the patients who met at least one of these criteria had better treatment outcomes and progressed further along the HIV care continuum.

## Introduction

This manuscript highlights a high priority need to improve mental health care among people living with HIV (PLWH) in order to impact progress along the HIV care continuum [1]. Early diagnosis of HIV, initiation of ART, and resulting viral suppression have been associated with improved immunologic function, fewer opportunistic infections, and longer life expectancy [2, 3]. Not only does the patient benefit from viral suppression, but the risk of transmission to partners in both heterosexual and homosexual relationships is markedly reduced [4, 5].

While global efforts aspire to improve progression along the HIV care continuum and end the HIV epidemic [6], there are many factors that complicate treatment. For some people living with HIV (PLWH), one of these barriers is mental health disease (MHD). Comorbid MHD is more commonly seen in both people vulnerable to acquiring HIV and PLWH compared to the general population [7]. It is estimated that 63% of PLWH have comorbid MHDs, compared to 31% of people without HIV. Depression is the most common comorbid MHD, affecting 20–40% of PLWH, and it has been shown to correlate with missed clinical appointments, virologic failure, and lack of retention in care [8]. PLWH also have higher rates of generalized anxiety disorder, affecting 16% of PLWH compared to 2% of the general population, in addition to a higher risk of developing schizophrenia and acute psychosis [8]. Because of the correlation between HIV and MHDs as well as the effects of MHD on progression along the HIV care continuum, thorough investigations and interventions are needed to improve the health outcomes for PLWH with comorbid MHD.

Previous studies have shown great benefits in providing access to mental health care while continuing treatment on the HIV care continuum [8]. While retention in care for mental health diagnoses can be as challenging as maintaining retention in care for HIV, it is not clear if even limited exposures to mental health care in a safety-net hospital system can show benefits in retaining patients throughout their treatment of HIV. To address this gap in knowledge, we performed a retrospective cohort study of all PLWH and comorbid MHD in the Adult Special Care Center (ASCC) in Memphis, Tennessee, from May 1, 2021, through May 1, 2023 to assess HIV care outcomes of PLWH with comorbid MHD who attended at least one clinic visit with a mental health care provider.

## Methods

All patients who attended at least one clinic visit at the ASCC between 5/1/2021 and 5/1/2023 were available for possible inclusion in the study. ASCC is a Ryan White-funded clinic serving over 2,000 PLWH annually during the study period and is part of the Regional One Health System, caring for a primarily underserved population in the metropolitan Memphis area in Tennessee. The clinic receives Part A, B, C, and D funding from the Ryan White program. The data was accessed in June 2023 and all patients who attended at least one clinic visit with a mental health provider working at ASCC, including a mental health counselor, psychiatric nurse practitioner, or psychiatrist, were included for manual chart review. These mental health providers receive a stipend from the Ryan White grant to provide mental health care within the ASCC, and patients are able to schedule appointments with their mental health providers independently from their medical providers. Patient demographics, laboratory data, clinic visit history, and HIV outcomes (retention in care and viral suppression) were abstracted from the EHR. This work was reviewed and approved by the University of Tennessee Health Science Center institutional review board and Regional One Health Office of Medical Research before any research activities were performed.

The primary outcomes of retention in care (attending two HIV clinic appointments 90 days apart within a 12-month span) and viral suppression (viral load of < 200 copies/mL) were defined in concordance with CDC definitions [9]. Both outcomes were assessed for the 12-month period immediately preceding and the 12-month period immediately following the first attended mental health clinic visit. If a patient was new to the clinic and did not have a 12-month period prior to their first attended mental health clinic visit, the 12-month prior data for retention in care and viral suppression were omitted and treated as missing data in all analyses.

All statistical tests were performed using SAS software, version 9.4 (SAS Institute, Cary, NC). A descriptive analysis of baseline characteristics was performed first. A multivariable logistic regression, including predetermined variables, was then performed to assess factors affecting viral suppression at 12 months after the first attended mental health clinic visit.

## Results

Patient demographics are described in Table 1. There were 174 PLWH who attended at least one mental health provider clinic visit between May 1, 2021, through May 1, 2023. The mean age was 43 years and 135 (78%) identified as Black. Of these, 103 (59%) identified as men, 63 (36%) as women, 7 (4%) as transgender women, and 1 (0.57%) as gender nonbinary. Substance use was common, with the most prevalent substances being used including tobacco in 116 (67%), marijuana in 100 (57%), and cocaine in 41 (24%) patients.

**Table 1:** Patient demographics, *N* = 174

Characteristic	Result
Age, years, mean (SD)	43 (12.07)
Gender, N (%)	
Male	103 (59.20)
Female	63 (36.21)
Transgender woman	7 (4.02)
Gender nonbinary	1 (0.57)
Race, N (%)	
Black	135 (77.59)
White	37 (21.26)
Other	2 (1.15)
Hispanic ethnicity, N (%)	2 (1.16)
Substance use, N (%)	
None	33 (18.97)
Tobacco	116 (66.67)
Marijuana	100 (57.47)
Cocaine	41 (23.56)
Methamphetamines	14 (8.05)
Opiates	8 (4.60)
Injection drug use	5 (2.87)
Polysubstance use, N (%)	89 (51.15)
Comorbid health conditions, N (%)	
None	89 (51.15)
Hypertension	67 (38.51)
Chronic lung disease	22 (12.64)
Diabetes mellitus	18 (10.34)
Chronic heart disease	9 (5.17)
Chronic kidney disease	8 (4.60)
Malignancy	6 (3.45)
Antiretroviral therapy, N (%)	
None	2 (1.15)
INSTI	140 (80.46)
PI	7 (4.02)
NNRTI, not EFV	4 (2.30)
EFV	1 (0.57)
Multiple	20 (11.49)
Mental health diagnoses, N (%)	
Depression	97 (55.75)
Anxiety	72 (41.38)
Bipolar disorder	36 (20.69)
Trauma or PTSD	34 (19.54)
Schizophrenia	22 (12.64)
Other^1^	57 (32.76)
History of psychiatric hospitalization, N (%)	18 (10.34)
Psychiatric medications, N (%)	
None	15 (8.62)
SSRI	80 (45.98)
Antipsychotics	70 (40.23)
SNRI	22 (12.64)
Benzodiazepines	5 (2.87)
Other^2^	113 (64.94)
Number of MH visits, median (IQR)	2 (1, 3)
Retention in care^3^, N (%)	
Prior to first MH visit, N = 168	139 (82.74)
After first MH visit, N = 151	131 (86.75)
Virally suppressed^4^, N (%)	
Prior to first MH visit, N = 167	96 (57.49)
After first MH visit, N = 135	108 (80)

Abbreviations SD, standard deviation; NNRTI, nonnucleoside reverse transcriptase inhibitor; EFV, efavirenz; PI, protease inhibitor; INSTI, integrase strand transfer inhibitor; PTSD, post traumatic stress disorder; SSRI, selective serotonin reuptake inhibitor; SNRI, serotonin and norepinephrine reuptake inhibitor; MH, mental health; IQR, interquartile range

^1^ Other diagnoses included attention deficit hyperactivity disorder, panic attacks, obsessive compulsive disorder, sleep disorders, Tourette’s disorder, agoraphobia, substance-induced mood disorder, delusional disorder

^2^ Other medications included hydroxyzine, tricyclic antidepressants, trazodone, mirtazapine, bupropion, buspirone, valproic acid, lamotrigine, and lithium

^3^ Retention in care was defined as attending two clinic visits 3 months apart in the 12 months prior to and 12 months after the patient’s first mental health visit. *p* = 0.36

^4^ Virally suppressed was defined as a viral load of < 200 copies/mL 12 +/- 3 months prior to and 12 +/- 3 months after the patient’s first mental health visit. *p* < 0.0001 by Chi-squared test

Comorbid mental health diagnoses included depression in 97 (56%), anxiety in 72 (41%), bipolar in 36 (21%), trauma/post-traumatic stress disorder (PTSD) in 34 (20%), and schizophrenia in 22 (13%) of patients. Other diagnoses not listed in the table included attention deficit hyperactivity disorder, panic attacks, obsessive compulsive disorder, sleep disorders, Tourette’s disorder, agoraphobia, substance-induced mood disorder, and delusional disorder. Many patients were diagnosed with multiple MHDs, and this is reflected in the percentages in Table 1. The most commonly used psychiatric medications include selective serotonin reuptake inhibitors (SSRI) in 80 (46%), antipsychotics in 70 (40%), and serotonin and norepinephrine reuptake inhibitor (SNRI) in 22 (13%) of patients. Other medications were used in 113 (65%) of patients and included hydroxyzine, tricyclic antidepressants, trazodone, mirtazapine, bupropion, buspirone, valproic acid, lamotrigine, and lithium. The median number of mental health visits per patient within the study period was two.

Regarding HIV care outcomes, 139/168 (83%) patients were retained in care in the 12 months prior and 131/151 (87%) in the 12 months following their first mental health clinic visit, which was not significantly different (*p* = 0.36). Additionally, 96/167 (57%) patients were virally suppressed 12 months prior and 108/135 (80%) were virally suppressed 12 months following their first mental health clinic visit, which was a significant difference when testing via univariate Chi-squared test (*p* < 0.0001).

Results from the multivariable logistic regression are shown in Table 2. Controlling for other variables, older age and having more mental health visits were significantly associated with higher rates of viral suppression.

**Table 2:** Factors affecting viral suppression after connection to a mental health provider (*N* = 135)

Characteristic	Not suppressed (*N* = 27)	Suppressed (*N* = 108)	*p* value^1^
Older Age, mean (SD)	40 (9.31)	46 (12.49)	0.03
Polysubstance abuse, N (%)	17 (62.96)	54 (50)	0.43
History of psychiatric hospitalization, N (%)	2 (7.41)	10 (9.26)	0.32
Number of MH visits, median (IQR)	2 (1, 2)	3 (1, 4)	0.03

Abbreviations SD, standard deviation; MH, mental health; IQR, interquartile range

^1^ Calculated by multivariable logistic regression, controlling for the other variables

## Discussion

In this retrospective cohort study evaluating PLWH with comorbid MHD in Memphis, TN, we observed significantly higher rates of viral suppression 12 months after attending a first mental health clinic visit in univariate analysis. We also found significantly higher rates of viral suppression among PLWH who were older and had more mental health clinic visits in our multivariable analysis.

While our findings support the idea that “some is good, but more is better,” it is important to not let perfection be the enemy of the good. While the multivariable analysis found significantly higher rates of viral suppression with increased numbers of mental health clinic visits, it is notable that even attending just one mental health clinic visit was found to be associated with higher rates of viral suppression in the univariate analysis. This is an important detail in addressing the shortage of mental health providers in the United States, where it is estimated that close to half of Americans live in a an area facing a mental health workforce shortage [10]. Previous works have shown poorer mental health outcomes in areas with lower access to mental health providers [11, 12]. Regarding HIV specifically, other works have highlighted the importance of mental health care in improving HIV outcomes [8, 13, 14]. Our work adds to these findings by showing that even attending just one mental health clinic visit was associated with improved rates of viral suppression. This is important because even in this clinic with in-house mental health providers, fewer than 10% of patients attended a mental health visit. Further research investigating barriers to receiving mental health care, including patient and provider-level factors, is needed. In a safety-net hospital system where mental health clinic visits are scarce, knowing that even just one mental health clinic visit improves HIV care outcomes is crucial.

By including a variety of mental health providers in our analysis, including licensed counselors, psychiatric nurse practitioners, as well as psychiatrists, our results support other works illustrating the benefits of counselors in addition to psychiatrists [15]. With so many people in the United States lacking access to mental health care providers [10], our results showing improved HIV care outcomes from any amount of contact with any mental health provider is encouraging.

Our work had several limitations. Due to limitations in accessing the clinic data, we were unable to include PLWH with documented MHD who never attended at least one mental health clinic visit or attended mental health services outside of the Regional One Health system, of which ASCC is a part. While we did assess retention in care and HIV viral suppression 12 months before and 12 months after the first mental health clinic visit, this limitation could lead to a selection bias if people who attend mental health clinic visits are more likely to respond to mental health care. We also had relatively small numbers, making subgroup analyses for specific mental health conditions or interventions (pharmacologic or counseling) limited. Finally, as a retrospective observational study, we are not able to control for unmeasured confounders or determine causality.

Overall, our work supports efforts to increase access to mental health care, particularly among vulnerable populations and PLWH [7, 16, 17]. While we found that attending even one mental health clinic visit significantly improved rates of viral suppression at one year, attending more visits further improved viral suppression rates. Further work should be done to evaluate which patients benefit most from different mental health interventions, including who needs long-term mental health follow-up care.
